# Portal vein thrombosis complicating neonatal umbilical vein catheterization in a 3-month-old infant with coincidental extrahepatic biliary atresia: A case report

**DOI:** 10.1097/MD.0000000000041238

**Published:** 2025-01-31

**Authors:** Mortada H.F. El-Shabrawi, Ayman Hussein Abdel Sattar, Fetouh Hassanin, Maha Fathy Sheba, Ahmed Elhennawy, Naglaa M. Kamal, Mohammed A.M. Oshi, Ali Algarni, Seham Anwar Emam Marzouk

**Affiliations:** a Professor of Pediatrics, Faculty of Medicine, Cairo University, Cairo, Egypt; b Professor of Pediatric Surgery, Faculty of Medicine, Cairo University, Cairo, Egypt; c Professor of Pediatrics, Faculty of Pharmacy, Misr International University, Cairo, Egypt; d Professor of Pathology, Faculty of Medicine, Cairo University, Cairo, Egypt; e Neurology Division, Pediatric Department, Gaafar Ibnauf Children’s Emergency Hospital, Khartoum, Sudan; f Department of Pediatrics, Taif Children Hospital, Taif, Saudi Arabia; g Lecturer of General Surgery, Faculty of Medicine, Beni Suef University, Beni Suef, Egypt.

**Keywords:** extrahepatic biliary atresia, kasai portoenterostomy, portal vein thrombosis

## Abstract

**Rationale:**

Umbilical vein catheterization (UVC) is a common procedure in neonatal intensive care units (NICU) but carries risks of severe complications such as portal vein thrombosis (PVT). Extrahepatic biliary atresia (EHBA), a leading cause of neonatal cholestasis, often progresses to end-stage liver disease. This case report discusses the rare coexistence of PVT and EHBA in a 3-month-old infant, highlighting the critical need for timely diagnosis and intervention.

**Patient concerns:**

A 3-month-old female presented with jaundice, dark-colored urine, and clay-colored stools. She had a history of NICU admission for neonatal sepsis during which a UVC was inserted.

**Diagnoses:**

Physical examination revealed jaundice and hepatosplenomegaly. Abdominal ultrasonography identified hepatosplenomegaly, mild ascites, and portal cavernoma. A liver biopsy confirmed a diagnosis of EHBA.

**Interventions:**

The patient underwent Kasai portoenterostomy. Postoperatively, she developed complications including ascites, systemic hypertension, and hyperammonemia. Initial improvements were observed with decreased bilirubin levels.

**Outcomes:**

Despite initial stabilization, the patient’s condition deteriorated, and she succumbed on day 15 postoperation.

**Lessons:**

This case underscores the significant risks of PVT associated with UVC and the importance of monitoring NICU graduates for early detection of complications. The early onset of portal hypertension and esophageal varices in this case challenges existing beliefs about EHBA’s clinical progression. Greater awareness and routine follow-up imaging are essential to improve outcomes in similar scenarios.

## 1. Introduction

The most common cause of vascular thrombosis in newborns is the use of central catheters.^[1]^ Neonatal portal vein thrombosis (PVT) is a rare event, occurring in approximately 1 in 100,000 live births,^[2]^ but is more frequently reported among graduates of neonatal intensive care units (NICU). PVT typically presents with portal hypertension (PH) and bleeding esophageal varices later in infancy or toddlerhood. Extrahepatic biliary atresia (EHBA) is a major cause of neonatal end-stage liver disease. Without timely diagnosis and treatment, most children with EHBA will develop irreversible liver fibrosis and PH within the first few months of life.^[3]^ To the best of our knowledge, coincidental PVT and EHBA have not been reported except after liver transplantation or Kasai portoenterostomy (KPE).

## 2. Case presentation

A 3-month-old female presented with jaundice, dark-colored urine, and clay-colored stools. She was born full term with a birth weight of 3.1 kg to a consanguineous, healthy couple. There was no maternal illness during pregnancy. The infant had a history of NICU admission for 10 days due to confirmed neonatal sepsis causing respiratory distress. An umbilical vein catheter (UVC) was inserted to provide the necessary treatment, which was frequently manipulated and repositioned.

**Figure 1.**
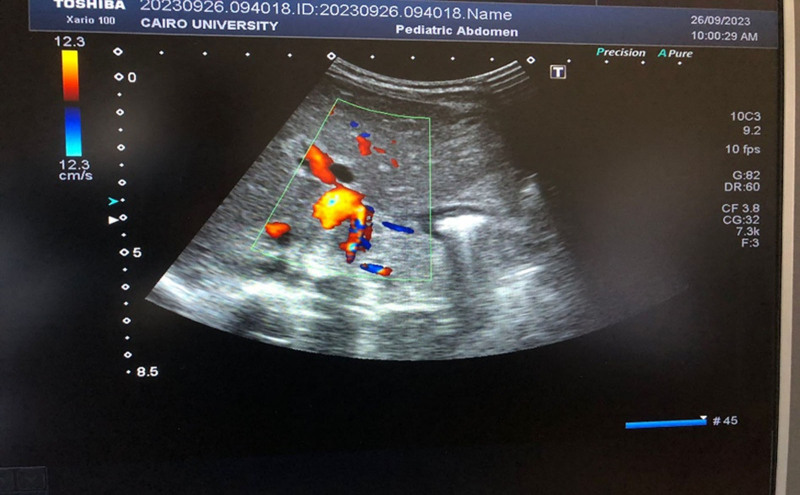
Color Doppler ultrasound scan showing portal cavernoma.

At 3 months of age, the infant weighed 4.4 kg and measured 53 cm in length. She was jaundiced and exhibited hepatosplenomegaly, with no other systemic abnormalities on examination. An abdominal ultrasound scan (USS) after a 4-hour fast revealed hepatosplenomegaly with mild ascites, and the gallbladder (GB) was not visualized. Color Doppler USS showed multiple collaterals at the site of the thrombosed portal vein, indicative of a portal cavernoma (Fig. 1).

**Figure 2.**
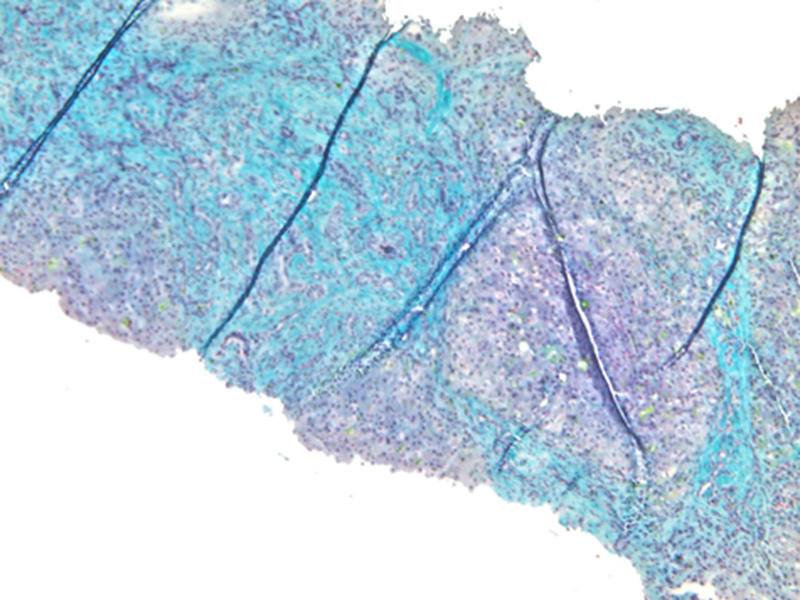
Needle liver biopsy showing distorted architecture, expanded portal areas with fibrous septa formation (Gomori trichrome, original magnification ×40).

A needle liver biopsy was performed, revealing distorted hepatic architecture and expanded portal areas with fibrous septa formation and proliferated bile ducts, some containing bile plugs. The hepatocytes exhibited mild ballooning, and there was evidence of intrahepatic and intracanalicular bile stasis (Fig. 2).

The patient underwent KPE. An intraoperative cholangiogram could not be performed through the atretic GB, confirming the diagnosis of EHBA. The procedure began with a rooftop incision followed by the mobilization and exteriorization of the liver through the release of the right and left triangular ligaments. The liver appeared cirrhotic, and an atretic GB was observed, along with ascitic fluid. At the porta hepatis, several peri-portal vein collaterals and a fibrotic main trunk (portal cavernoma) were noted. Lower peri-esophageal and gastric varices were also observed during the mobilization of the left lobe of the liver.

**Figure 3.**
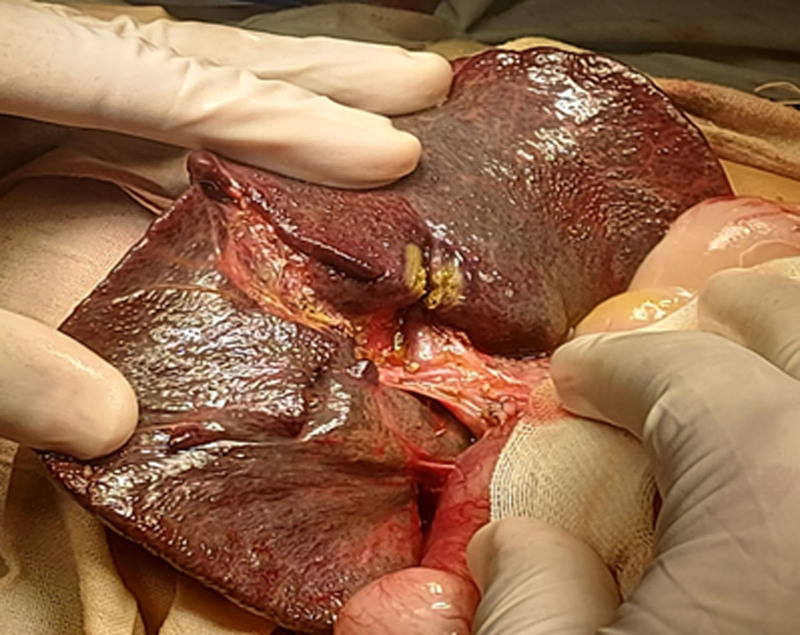
Intraoperative photo showing early cirrhotic liver and several peri-portal vein collaterals at the porta hepatis with a fibrotic main trunk (portal cavernoma).

The atretic GB was mobilized from its bed and excised along with the biliary remnants. The portal plate above the bifurcation of the portal vein, toward the corners of the hilar ductal plate, was meticulously dissected. The fibrous remnant in the ductal plate was excised using sharp dissection (Fig. 3). A Roux-en-Y jejuno-jejunal anastomosis was performed 20 cm from the duodeno-jejunal junction, with an ascending limb of 40 cm. The anastomosis was completed side-to-end with 2-layer interrupted sutures.

Pathological examination of the excised surgical specimen revealed a rudimentary GB with flattened mucosa and sparse chronic inflammatory infiltrate in the submucosa and muscle coat. The cystic duct and distal bile duct radicle showed marked lumen narrowing with sparse inflammatory infiltrate.

## 3. Postoperative course

Postoperatively, the infant was transferred to the NICU. Her course was turbulent; however, she passed pigmented stools on day 2 and started oral fluids on day 3. Her total bilirubin levels decreased from 16 to 9.5 mg/dL. On day 8, she developed ascites with a drain output of 800 mL/d and started experiencing unexplained systemic hypertension, necessitating the addition of amlodipine, spironolactone, and alpha methyldopa. On day 10, the drain slipped and had to be reinserted. By day 12, the patient became hypoactive, required high nasal flow oxygen, began passing clay-colored stools, and had an average drain output of 400 to 500 mL/d. Her ammonia levels were elevated to 82 µmol/L. She died on day 15.

## 4. Discussion

UVC in neonates can lead to several complications, including PVT, a significant cause of extrahepatic PH and upper gastrointestinal bleeding in children.^[4]^ EHBA is characterized by fibro-sclerosing obliteration of the extrahepatic bile ducts. Most infants with EHBA progress to chronic end-stage liver disease, PH, and its complications, such as massive bleeding from esophageal or gastric varices, ascites, hepato-renal syndrome, and hepatic encephalopathy.^[5]^ In this case, EHBA was associated with PVT and early PH with mild ascites. The French National Study reported a definite advantage of early KPE.^[6]^ Our 3-month-old patient developed early manifestations of PH, evidenced by esophageal varices detected during surgery, likely due to PVT induced by UVC. This challenges the notion that esophageal varices usually appear after 1 year of age.

The coexistence of EHBA with other congenital anomalies has been reported, including malrotation, liver asymmetry, situs inversus, absent inferior vena cava, preduodenal portal vein, and polysplenia.^[7]^ However, the association of PVT and EHBA in our patient exacerbated their pathological impacts.

## 5. Conclusion

PVT can occur as a complication of neonatal UVC. It is often asymptomatic in the neonatal period and not clinically recognizable but can be easily detected by duplex Doppler USS. We strongly recommend routine checking of UVC position immediately after placement and during hospitalization to prevent future PVT in NICUs. Neonatologists and pediatricians should be aware of this potential complication to ensure proper catheter placement. Additionally, we recommend follow-up of NICU graduates with abdominal USS to diagnose early PVT development.

## Acknowledgments

The authors thank their patient and his family.

## Author contributions

**Conceptualization:** Mortada H.F. El-Shabrawi, Fetouh Hassanin.

**Data curation:** Mortada H.F. El-Shabrawi, Fetouh Hassanin.

**Formal analysis:** Mortada H.F. El-Shabrawi, Fetouh Hassanin.

**Investigation:** Mortada H.F. El-Shabrawi, Ayman Hussein Abdel Sattar, Fetouh Hassanin, Maha Fathy Sheba, Ahmed Elhennawy, Seham Anwar Emam Marzouk.

**Methodology:** Mortada H.F. El-Shabrawi, Ayman Hussein Abdel Sattar, Fetouh Hassanin, Maha Fathy Sheba, Ahmed Elhennawy, Seham Anwar Emam Marzouk.

**Supervision:** Mortada H.F. El-Shabrawi.

**Validation:** Mortada H.F. El-Shabrawi.

**Visualization:** Mortada H.F. El-Shabrawi.

**Writing—original draft:** Mortada H.F. El-Shabrawi, Fetouh Hassanin, Naglaa M. Kamal, Mohammed A.M. Oshi, Ali Algarni.

**Writing—review & editing:** Mortada H.F. El-Shabrawi, Ayman Hussein Abdel Sattar, Fetouh Hassanin, Maha Fathy Sheba, Ahmed Elhennawy, Naglaa M. Kamal, Mohammed A.M. Oshi, Ali Algarni, Seham Anwar Emam Marzouk.

**Project administration:** Ayman Hussein Abdel Sattar, Maha Fathy Sheba, Ahmed Elhennawy, Seham Anwar Emam Marzouk.
